# A high-resolution gridded grazing dataset of grassland ecosystem on the Qinghai–Tibet Plateau in 1982–2015

**DOI:** 10.1038/s41597-023-01970-1

**Published:** 2023-02-02

**Authors:** Nan Meng, Lijing Wang, Wenchao Qi, Xuhuan Dai, Zuzheng Li, Yanzheng Yang, Ruonan Li, Jinfeng Ma, Hua Zheng

**Affiliations:** 1 State Key Laboratory of Urban and Regional Ecology, Research Center for Eco-Environmental Sciences, Chinese Academy of Sciences, Beijing, 100085 China; 2 University of Chinese Academy of Sciences, Beijing, 100049 China; 3 Aerospace Information Research Institute, Chinese Academy of Sciences, Beijing, 100094 China

**Keywords:** Ecosystem ecology, Environmental economics

## Abstract

Grazing intensity, characterized by high spatial heterogeneity, is a vital parameter to accurately depict human disturbance and its effects on grassland ecosystems. Grazing census data provide useful county-scale information; however, they do not accurately delineate spatial heterogeneity within counties, and a high-resolution dataset is urgently needed. Therefore, we built a methodological framework combining the cross-scale feature extraction method and a random forest model to spatialize census data after fully considering four features affecting grazing, and produced a high-resolution gridded grazing dataset on the Qinghai–Tibet Plateau in 1982–2015. The proposed method (R^2^ = 0.80) exhibited 35.59% higher accuracy than the traditional method. Our dataset were highly consistent with census data (R^2^ of spatial accuracy = 0.96, NSE of temporal accuracy = 0.96) and field data (R^2^ of spatial accuracy = 0.77). Compared with public datasets, our dataset featured a higher temporal resolution (1982–2015) and spatial resolution (over two times higher). Thus, it has the potential to elucidate the spatiotemporal variation in human activities and guide the sustainable management of grassland ecosystem.

## Background & Summary

Grazing is a direct and critical indicator of anthropogenic interference on grasslands^1^, and the high-spatiotemporal-resolution grazing datasets have been indispensable tools for investigating the influence of grazing on grassland ecosystems^2^. Detailed geographic grazing information, despite its importance, is not readily available, which is the most pressing challenge limiting grassland ecosystem management. For example, the grassland degradation^3,4^, caused by the human demand for meat and milk production^5^ and inappropriate grazing management^6,7^, is increasingly severe, which is bound to exacerbate human and wildlife conflict because of the aggravated resource competition^8^. The poor spatial heterogeneity of census grazing data makes it difficult to be combined with remote sensing data for analysis. Therefore, the mechanism of the effect of spatio-temporal changes of grazing on grassland degradation has been rarely documented^9,10^, which limits the understanding of the influence of grazing on grassland degradation and coordinate the underlying conflicts between livestock production and wild herbivore conservation^11^. There is an urgent need to obtain reliable high-spatiotemporal-resolution grazing datasets to accurately depict spatially explicit and temporally explicit changes, particularly for fragile grassland ecosystems with a significant contradiction between economic development and sustainable development.

Clarifying the factors influencing the spatial preferences of grazing is vital for obtaining high-spatiotemporal-resolution grazing datasets, considering the inconsistent drivers emerged in producing previous datasets^12,13^. Grazing, a kind of animal husbandry production activity, is a grassland management and utilization mode^14^. Climatic and environmental factors, herds’ foraging behavior, herders’ grazing behavior, and ecological protection policies influence the spatial heterogeneity of the distribution of livestock grazing. For example, climatic (i.e., temperature and precipitation) and environmental factors (i.e., terrain slope and soil type) restrict the spatial distribution of livestock^15–17^. The foraging behavior of herds has further influence, for instance, grazing hotspots have also been observed near areas with sufficient water resources and pasture^18,19^. Moreover, the grazing behaviors of herders are also often closely linked to the travel characteristics of livestock, because the animals move back and forth near herders’ settlements^20^. Several studies have also reported that ecological protection policies, such as natural reserves, influence resource utilization and human–nature relationship, and have notable restriction for grazing distribution according to the conservation level^21^. Therefore, it is crucial to further investigate how to fully leverage the spatiotemporal heterogeneity of the influencing factors to more reasonably produce high-resolution gridded grazing datasets.

The method for obtaining high-spatiotemporal-resolution grazing datasets for large-scale areas is also important. The current methods can be broadly classified into two categories: The first category of methods are the simulation methods based on remote sensing data, such as grassland utilization intensity^22^ and the human appropriation of net primary production^23^. These methods are substitute indexes of grazing intensity obtained through the simulation of the difference between the potential and real vegetation states, and they are considered to be equivalent to the grazing intensity in grassland ecosystems. Although it is convenient to obtain the spatial heterogeneity of grazing at the pixel level, it may be a challenge for areas with animal husbandry coexisting with abundant herbivorous wildlife, because eliminating the impact of herbivorous wildlife is difficult^24^. The second category of methods are the spatialization methods based on census grazing data. These methods construct the administrative-level relationship between grazing density and influencing factors using multiple linear regression^12^ or machine learning algorithms^13,25^, and then, gridded grazing density is obtained the using the trained model and pixel-level influencing factors. The methods are characterized by grazing data accuracy; however, a scale mismatch exists when the carrier of grazing information is transformed from the administrative level to the pixel level, because the trained model fails to accurately capture the extreme values of the predicted data accurately. Hence, how to integrate the advantages of the two methods to obtain a gridded grazing dataset with both high spatial heterogeneity and high accuracy remains a challenge.

The combination of cross-scale features (CSFs) extraction method and machine learning algorithm can be applied to solves these key issues. The combined approach can integrate the advantages of the two data production methods. The CSFs extraction method can narrow the scale differences in feature representation between administrative-level and pixel-level by replacing the average administrative-level value with a finer-level value, thereby increasing the training sample size and range and ameliorating the scale mismatch problem in the relationship transmission process^26^. Additionally, the random forest (RF) model, a widely used and well-understood non-parametric machine learning algorithm for regression analysis, is characterized by high computational efficiency and estimation accuracy, and has been used in the spatialization of various census data owing to its simplicity^25,27,28^. Most important, taking the extracted CSFs as the training data of RF (RF–CSF) model, the advantage of high spatial heterogeneity in the former method and high accuracy of census data in the latter method can be effectively integrated to reduce cross scale differences, and achieve accurate transmission of feature information. In addition to the above advantages, the RF–CSF model has also preponderance in integrating the driving forces of grazing distribution. For example, the RF model can be used to calculate the importance of each independent variable^29^ and simulate the complex nonlinear relationship between multidimensional independent variables and the dependent variable^30^, which meets our requirements for integrating the characteristics of multi-source factors.

Qinghai-Tibet Plateau has a more urgent and realistic need to obtain a high-spatiotemporal-resolution grazing dataset to mitigate grassland degradation and enhance sustainability through grazing intensity adjustment^31^, because the Qinghai-Tibet Plateau is a typical animal husbandry area with fragile ecosystem and has undergone continuous aggravated grassland degradation over the past few decades^32,33^. Therefore, the objectives of the present study are (i) to build a methodological framework to improve the traditional methods for developing gridded grazing datasets, (ii) to produce a high-resolution gridded grazing dataset of grassland ecosystems on the Qinghai–Tibet Plateau for 1982–2015, and (iii) to validate the accuracy of the dataset. The dataset can be applied to elucidate the temporal dynamics and spatial variation of human disturbance and their influences on grasslands, and it can also be used in other related studies on the Qinghai–Tibet Plateau.

## Methods

### Study area

**Fig. 1:**
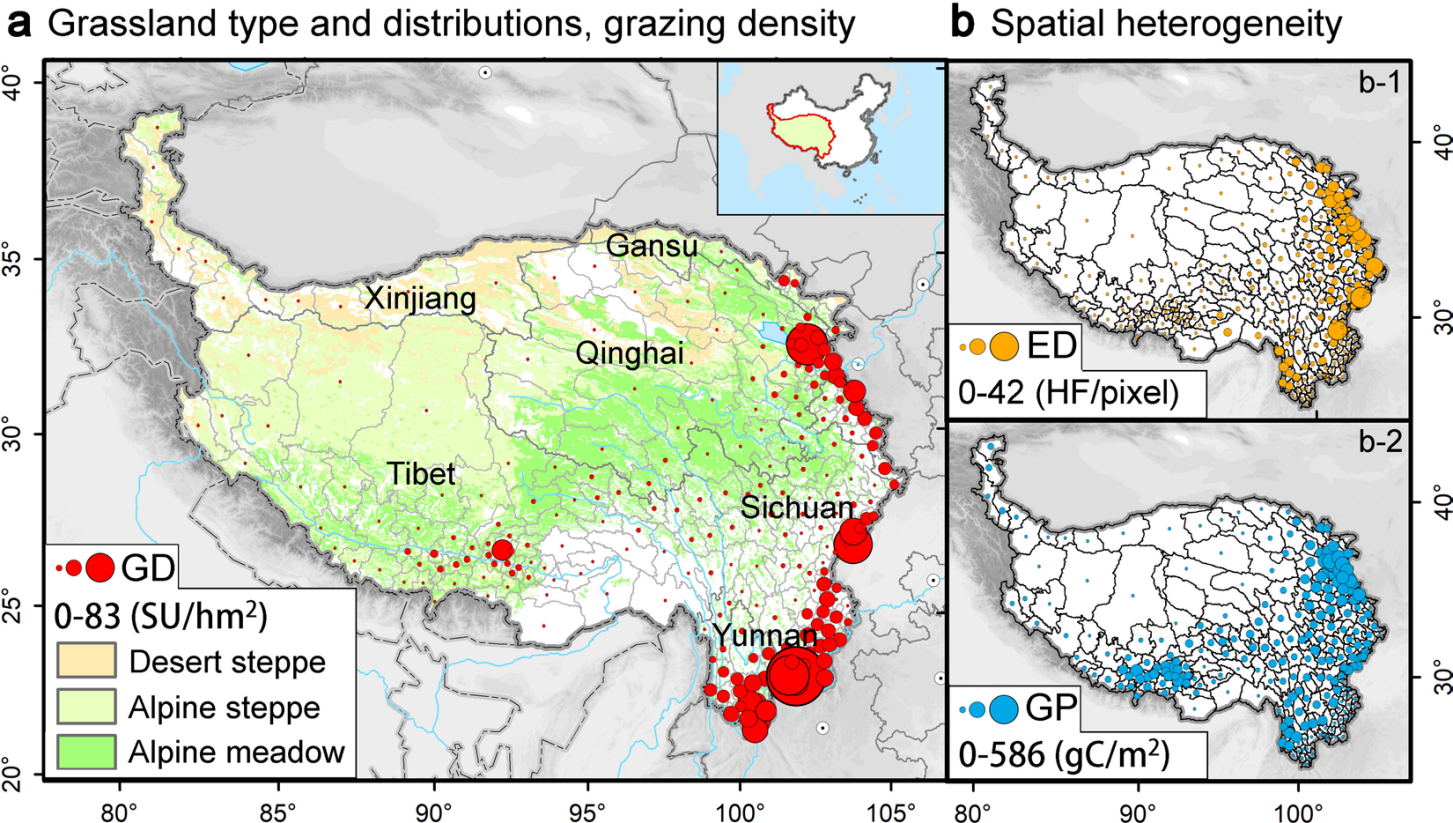
Location of the Qinghai–Tibet Plateau: (**a**) grassland type and distribution, and grazing density (GD) in 244 counties; (**b**) spatial heterogeneity of economic development (ED) and grassland production (GP) in 244 counties. GD, ED, and GP are represented by sheep unit per grassland area per county (SU/hm^2^), human footprint index per pixel (HF/pixel) per county, and net primary production per grassland area per county (gC/m^2^), respectively.

**Fig. 2:**
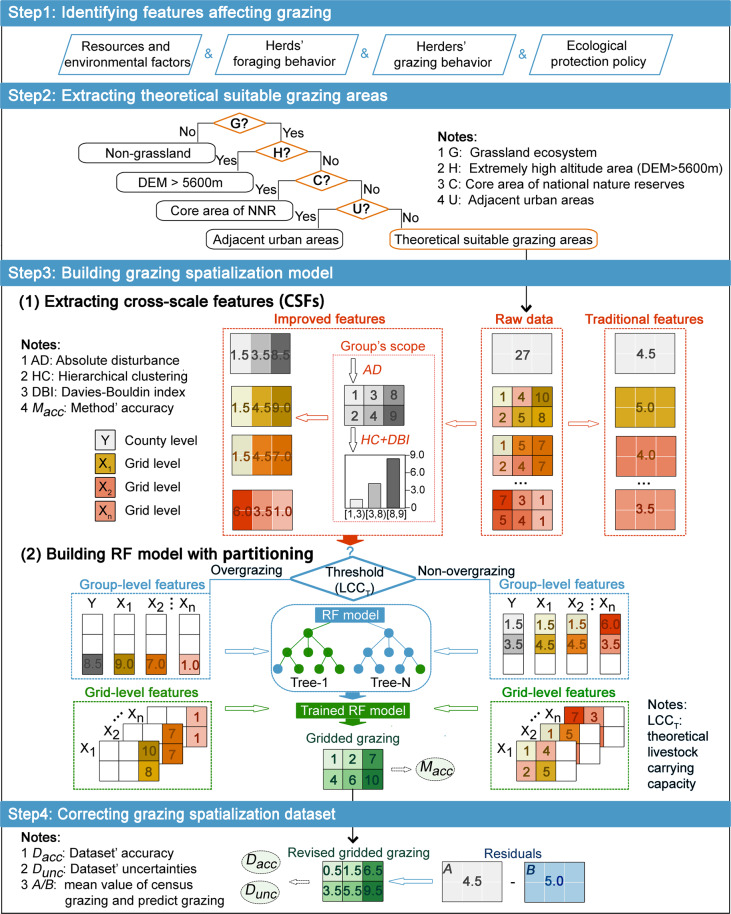
Methodological framework for grazing spatialization.

The Qinghai–Tibet Plateau (26°00′-39°47′N, 73°19′-104°47′E), one of the most important pastoral areas in the world, straddles the southwest regions of China, and it includes 244 counties, which belong to six provinces: Tibet, Qinghai, Xinjiang, Gansu, Sichuan, and Yunnan. It is characterized by rich natural grassland resources, including desert steppes, alpine steppes, and alpine meadows (Fig. 1a). The grassland areas account for over 56% of this region^34^. The grassland plays a vital role in providing regional and national animal husbandry products and fodder^35^, which enables the local herders to obtain almost all of the resources required for survival^36^. The grazing density distribution is extremely unbalanced (Fig. 1a) owing to the high spatial heterogeneity of economic development (Fig. 1b-1) and grassland production (Fig. 1b-2), resulting from the differences in resources and environmental factors^37^. Over the past few decades, there has been a significant change in the number of livestock animals, and the number of sheep exceeded 160 million by 2020. Therefore, it is urgent to obtain a high-resolution gridded grazing dataset for its evaluating spatiotemporal changes and coordinating the relationship between human beings and the grassland ecosystem.

### Methodological framework

We developed a methodological framework for high-resolution gridded grazing dataset mapping. The framework mainly includes four parts: (i) identifying features affecting grazing, (ii) extracting theoretical suitable grazing areas, (iii) building grazing spatialization model, and (iv) correcting the grazing spatialization dataset. Each step is explained in more detail below (Fig. 2).

### Step 1: Identifying features affecting grazing

Grazing activities are affected by the spatial heterogeneity of resources and environmental factors, regulated by the grazing behavior of herders and the foraging behavior of herds, and restricted by ecological protection policies. Therefore, the specific implications of the 14 influencing factors from the above four aspects are presented in Table 1. These factors are necessary for spatializing the county-level grazing data.

**Table 1:** The identified features affecting grazing.

Types	Descriptions and Implications	Variables
Resources and environmental factors	Climate and soil texture significantly affect vegetation growth^53^.	Precipitation
		Temperature
		Radiation
		Soil moisture
		Soil pH
		Soil available N
		Soil available P
		Soil available K
Herders’ grazing behavior	High-coverage grasslands close to residential areas and water sources are more convenient for herders to graze^20^.	NDVI
		Distance to river
		Residential area density
Herds’ foraging behavior	Herds prefer to feed on grassland with high suitability and easy access^20^.	Pasture suitability
		Topographic relief
Ecological protection policy	Protected areas with different protection levels were formulated to combat grassland degradation^12^.	Nature reserves

### Step 2: Extracting theoretical suitable grazing areas

The decision tree approach^38^ was adopted to extract the theoretical suitable grazing areas for further grazing spatialization (step 2 in Fig. 2). First, the potential grazing area was identified according to the boundary of the grassland ecosystem, because grazing behavior only occurs in the grassland. Then, the unsuitable areas for grazing, i.e., extremely-high-altitude areas and areas adjacent to towns, were removed from the potential grazing area stepwise. The areas strictly prohibited for grazing, i.e., the core areas of national nature reserves^39^ within grassland areas, were also deemed unsuitable for grazing. Finally, the extracted areas were the theoretically suitable grazing areas.

### Step 3: Building grazing spatialization model

#### (i) Extracting cross-scale feature (CSFs)

In the traditional method, the spatial resolution of the training data (i.e., the average value at the administrative level) differs from that of the predicting data (i.e., the value at the pixel level), and the trained model can only capture the characteristics within the training data. However, the extreme value of the predicting data inevitably exceeds the range of the training data, which can result in underestimation in these parts^40^. To reduce these mismatches, we built an improved method for CSFs extraction (Fig. 2, first part of step 3).

First, the census grazing data are simply distributed from county level to pixel level using the weight of the absolute disturbance (AD) index as Eq. (1). The AD index is measured by Mahalanobis distance using Eq. (2), which is calculated according to the deviation between the potential and observed normalized difference vegetation index (NDVI) values^22^. Second, the distributed grazing data are graded via the hierarchical clustering method, and the optimal number of the group can be determined using the Davies–Bouldin index (DBI)^41^ as Eq. (3), an index for evaluating the quality of clustering algorithm. The smaller the DBI, the smaller the distance within each group. Therefore, the DBI can be used to select the best similar values to minimize the deviation within each group. Finally, we can obtain the scope of the groups within each county using the above two steps and obtain the average value of all independent variables and the dependent variable accordingly. As expected, we can decompose the average value at the county level (traditional features in Fig. 2) into the average value at the group level (improved features in Fig. 2).

1 $$S{U}_{i}=S{U}_{j}^{C}\frac{{w}_{A{D}_{i}}}{{w}_{A{D}_{j}}}$$

where *SU*_*i*_ and $S{U}_{j}^{C}$ are the grazing value for pixel i and the census grazing value for county j; ${w}_{A{D}_{i}}$ is the weight of the AD index for pixel i and ${w}_{A{D}_{j}}$ represents the summed weight of the AD index values for all pixels in county j.

2 $$\begin{array}{cll}A{D}_{i} & = & \sqrt{{({D}_{i}-u)}^{T}co{v}^{-1}({D}_{i}-u)}\\ {D}_{i} & = & NDV{I}_{i}^{T}-NDV{I}_{i}^{P}\end{array}$$

where *AD*_*i*_ is the AD index for pixel i; the vector composed of its observed NDVI $\left(NDV{I}_{i}^{T}\right)$ and potential NDVI $\left(NDV{I}_{i}^{P}\right)$ time-series data could be considered as two points in the feature space for pixel i, and *D*_*i*_ and *u* are the difference and the mean value of the vector, respectively; *cov* is the covariance matrix.

3 $$DB{I}_{k}=\frac{1}{k}{\sum }_{x=1}^{k}ma{x}_{y\ne x}\left(\frac{\overline{{a}_{x}}+\overline{{a}_{y}}}{\left|{\delta }_{x}-{\delta }_{y}\right|}\right)$$

where *DBI*_*k*_ is the DBI coefficient when the cluster number is *k*; $\overline{{a}_{x}}$ and $\overline{{a}_{y}}$ are the average distances of the group *x*_*th*_ and the group *y*_*th*_, respectively; *δ*_*x*_ and *δ*_*y*_ are the center distance of the group *x*_*th*_ and the group *y*_*th*_, respectively.

Different from the traditional method, our method can decompose features into multiple features using the grading AD index. The differences among counties will not be easily averaged out. Moreover, our method is less affected by scale mismatch and can be transferred to cross-scale modeling^26^.

#### (ii) Building RF model with partitioning

A single model cannot accurately obtain the variation information of the Qinghai–Tibet Plateau with high spatial heterogeneity. The partition model, a widely used method for estimating population distribution and others^42,43^, can be incorporated into the proposed model to improve its performance. The thresholds (0.43, 0.35 and 0.21 SU/hm^2^), determined according to the theoretical livestock carrying capacity (equation S1), were calculated and used to separate independent variables and dependent variable for each grassland types: alpine meadow, alpine steppe and alpine desert steppe (see Section 6.1 for details). Then, the RF models were established, and the training and testing samples were randomly divided in the proportion of 3:1. It is notable that transforming the response variable using natural log prior to RF model fitting is necessary to achieve higher prediction accuracies^44^. Finally, the independent variables at the pixel level were inputted into the two trained RF models, and the corresponding grid grazing dataset was output by combining the two results (Fig. 2, second part of step 3).

#### (iii) Validating the accuracy of the methods

The performance of the grazing spatialization model was evaluated through a comparison of the predicted value with census value^26^. Accuracy validation indexes, including coefficients of determination (R^2^), root mean square error (RMSE), and mean absolute error (MAE), were used to evaluate the performances of the proposed RF-based models (Table 2), as presented in Eq. (4).

4 $$\begin{array}{ccc}{R}^{2} & = & 1-\frac{{\sum }_{j=1}^{N}{\left(S{U}_{j}^{C}-S{U}_{j}^{P}\right)}^{2}}{{\sum }_{j=1}^{N}{\left(S{U}_{j}^{C}-\overline{S{U}^{C}}\right)}^{2}}\\ RMSE & = & \sqrt{\frac{{\sum }_{j=1}^{N}{\left(S{U}_{j}^{C}-S{U}_{j}^{P}\right)}^{2}}{N}}\\ MAE & = & \frac{{\sum }_{j=1}^{N}| S{U}_{j}^{C}-S{U}_{j}^{P}| }{N}\end{array}$$

where $S{U}_{j}^{C}$ and $S{U}_{j}^{P}$ are the census grazing value and the predicted grazing value for county j, respectively; $\overline{S{U}^{C}}$ is the average census data for all counties; and *N* is the number of all counties.

**Table 2:** The proposed methods and their descriptions.

Method	Description
Traditional RF model (M1)	An RF model in which the average value of all variables at the county level is used as the training sample.
RF model with a partition (M2)	Two RF models based on the threshold of the theoretical livestock -carrying capacity; the average value of all variables at the county level is used as the training samples.
RF model with CSFs (M3)	An RF model in which the average value of all variables at the group level (cross-feature extraction method) is used as the training sample.
RF model with partition and CSFs (M4)	The RF models, based on the threshold of the theoretical livestock-carrying capacity; the average value of all of the variables at the group level (cross-feature extraction method) is used as the training sample.

### Step 4: Correcting grazing spatialization dataset

#### (i) Correcting residuals of dataset

Correcting residuals is necessary to obtain datasets with higher accuracy^45,46^, because propagating the cross-scale relationship in the RF models will inevitably generate errors^47^. The residuals, calculated by the difference between the average census grazing and predicted grazing values at the administrative level, were used to calibrate the errors related to all pixels within this county. The revised dataset after residual correction is the final product provided in this study. The residual correction method is expressed by Eq. (5), and the process is shown in the fourth step in Fig. 2.

5 $$S{U}_{i}^{RP}=S{U}_{i}^{P}+{R}_{j}$$

where $S{U}_{i}^{RP}$ denotes the predicted grazing value revised by the residuals for pixel i, $S{U}_{i}^{P}$ denotes the predicted grazing for pixel i, and *R*_*j*_ denotes the residuals calculated from the difference between census grazing and predicted grazing data for county j.

#### (ii) Validating the accuracy of dataset

Two goodness-of-fit indexes were used to validate the consistency of spatial distribution and the temporal trend between predicted grazing data and census grazing data. Generally, the coefficient of determination (R^2^), defined in Eq. (4), is used to verify the consistency of spatial distribution, and the Nash–Sutcliffe efficiency (NSE, Eq. (6)) is used to verify the consistency of temporal trend. An index value closer to 1 corresponds to a more accurate dataset. Meanwhile, we also collected field grazing data from 56 sites to further validate the spatial accuracy of the dataset, and it measured using the R^2^ in Eq. (4).

6 $$NSE=1-\frac{{\sum }_{t=1}^{T}{\left(S{U}_{t}^{RP}-S{U}_{t}^{C}\right)}^{2}}{{\sum }_{t=1}^{T}{\left(S{U}_{t}^{C}-\overline{S{U}^{{C}^{{\prime} }}}\right)}^{2}}$$

where $S{U}_{t}^{RP}$ and $S{U}_{t}^{C}$ are the predicted grazing value revised by residuals and the census grazing value of all counties in year *t*, respectively; $\overline{S{U}^{{C}^{{\prime} }}}$ is the average census grazing value of all years; and *T* is the number of time steps.

#### (iii) Identifying uncertainties associated with dataset

The uncertainties associated with the dataset originate from the following two aspects: First, the unreasonableness of our method, owing to the errors related to cross-scale modeling or the inappropriate selection of influencing factors, is an important source of uncertainties. Second, the incompleteness of auxiliary variables also introduces uncertainties. In this instance, grassland-free areas are not accurately identified in some counties, but livestock animals are raised in these counties. These counties have no effective value for livestock density prediction. Overall, the uncertainties can be identified in terms of the mean relative error (MRE) in Eq. (7).

7 $$MRE=\frac{{\sum }_{j=1}^{N}\left|\frac{S{U}_{j}^{C}-S{U}_{j}^{RP}}{S{U}_{j}^{C}}\right|}{N}\ast 100 \% $$

where $S{U}_{j}^{C}$ is the census grazing value for county j, $S{U}_{j}^{RP}$ is the predicted grazing value revised by residuals for county j, and *N* is the number of counties.

### Data source

#### Census grazing data at county level

Eight types of livestock, namely cattle, yaks, horses, donkeys, mules, camels, goats, and sheep, were considered according to the regional characteristics, and livestock stocking quantity at the end of year for each county can be determined from statistical yearbooks. However, the numbers of livestock at the county level for some years between 1982 and 2015 were not recorded. The missing data were indirectly approximated from city- or provincial-level data (e.g., interpolation using their temporal trends). Each type of livestock stocking quantity was converted into standard sheep unit (SU) according to the national standards using Eq. (8)^48^, namely the calculation of rangeland carrying capacity (NY/T 635-2015). Of the 244 counties of the Qinghai–Tibet Plateau, only 242 counties were considered, as the census grazing data for the other 2 counties were unavailable. The unit of grazing statistics data at the county level is defined as SU per county per year (SU·county^−1^·year^−1^).

8 $$\begin{array}{l}SU={N}_{sheep}+0.8\times {N}_{goats}+5\times {N}_{cattle}+5\times {N}_{yaks+}+\\ 6\times {N}_{horses}+3\times {N}_{donkeys}+6\times {N}_{mules}+7\times {N}_{camels}\end{array}$$

where *N*_*sheep*_, *N*_*goats*_, *N*_*cattle*_, *N*_*yaks*_, *N*_*horses*_, *N*_*donkeys*_, *N*_*mules*_, *N*_*camels*_ are the number of sheep, goats, cattle, yaks, horses, donkeys, mules, and camels at the year-end, respectively. *SU* denotes the standard sheep unit (SU·county^−1^·year^−1^).

#### Data of grazing influencing factors at pixel level

The types of features affecting grazing were obtained from the first step described in Methods, and the detailed information, such as original spatiotemporal resolution, format, and source, is shown in Table 3. The format (i.e., GeoTIFF), spatial resolution (i.e., 0.083°), and the number of rows and columns of the gridded features were leveraged to further produce a high-resolution grazing dataset.

**Table 3:** Data source of grazing influence factors.

Variables	Temporal resolution	Spatial resolution	Original format	Data source
Precipitation	1982–2015	—	Textfile	https://data.cma.cn/
Temperature				
Radiation				
Soil moisture	2000–2015	0.25°	NetCDF	https://disc.gsfc.nasa.gov/
Soil pH	—	30 arc seconds	GeoTIFF	https://data.tpdc.ac.cn/zh-hans/^54,55^
Soil available N				
Soil available P				
Soil available K				
NDVI	1982–2015	0.083°	NetCDF	https://ecocast.arc.nasa.gov/data/pub/gimms/3g.v1/
Distance to river	2015	90 m	GeoTIFF	Institute of Remote Sensing and Digital Earth, Chinese Academy of Sciences
Residential area density	2015	—	Shapefile	https://data.tpdc.ac.cn/zh-hans/
Pasture suitability	2005	10 km	GeoTIFF	https://data.apps.fao.org/map/catalog
Topographic relief	2010	90 m	GeoTIFF	https://www.usgs.gov/
Nature reserves	2015	—	Shapefile	Collected from relevant government departments

## Data Records

The gridded grazing dataset of the grassland ecosystem on the Qinghai–Tibet Plateau for 1982–2015^49^ is available at 10.6084/m9.figshare.21501390. The dataset was named according to the year corresponding to the grazing data and stored in GeoTIFF format in SU per pixel per year. The spatial resolution was 0.083°, with an annual temporal resolution. The geographic coordinate system of the dataset was GCS_WGS_1984. To reduce the file size, the data of 34 years were compressed and stored in zip format. They can be downloaded, uncompressed, and then viewed using various GIS software programs.

## Technical Validation

### Performance of grazing spatialization method

**Fig. 3:**
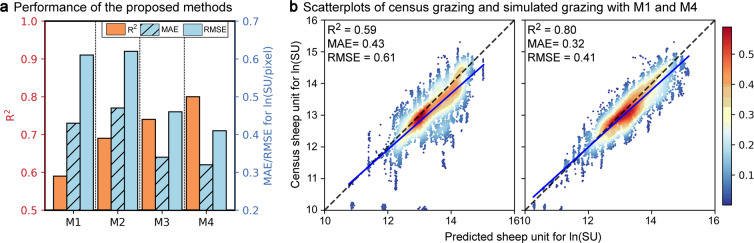
Performance of the proposed method on the sum of SUs for each county from 1982 to 2015: (**a**) Performances of the proposed methods. M1, M2, M3, and M4 denote the traditional RF model, RF model with a partition, RF model with CSFs, and RF model with a partition and CSFs, respectively. (**b**) Scatterplots of M1- and M4-predicted census grazing and predicted grazing. The blue solid line and the black dotted line are the fitting line and the 1:1 diagonal line, respectively.

Compared with the traditional RF model (M1), the proposed RF model with a partition and CSFs (M4) exhibited 35.59% higher overall accuracy. The proposed method considerably contributes to the accuracy of the traditional RF model (M1). Each step, including the RF model with partition (M2), RF model with CSFs (M3), and RF model with partition and CSFs (M4), improved the accuracy of the model (Fig. 3a). Compared with M1 (R^2^ = 0.59), M2 and M3 exhibited 0.10 and 0.15 higher R^2^, respectively, and reduced errors. Moreover, the overall R^2^ of M4 reached 0.80. Figure 3b shows the scatter plot and the fitting results between the census grazing data and the M1- and M4-predicted data for each county in 1982–2015. The M4 data exhibited a comparable scatter point distribution pattern to M1, but the scatter points were more convergent to the 1:1 diagonal line. The comparison of the R^2^ values of M1 and M4 reveals that M4 remarkably improved prediction accuracy; thus, M4 can alleviate scale mismatch in grazing spatialization.

### Validation of the grazing spatialization dataset at the county level

**Fig. 4:**
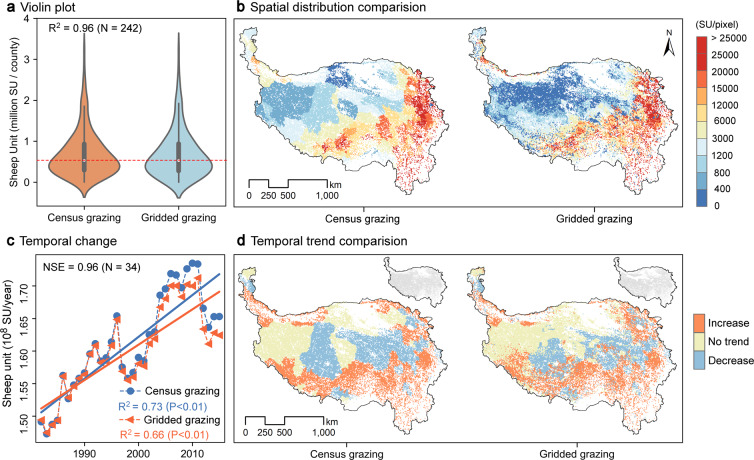
Validation of the grazing spatialization dataset using census grazing data: (**a**) violin plot of census grazing and gridded grazing in 2015; (**b**) spatial distribution in SU per pixel in 2015; (**c**) temporal change in 10^8^ SU per year from 1982 to 2015; (**d**) temporal trend in SU per pixel from 1982 to 2015, and the gray areas indicate significant threds (p < 0.05).

Our dataset was highly consistent with the county-level census grazing dataset, and the spatial accuracy (R^2^) was 0.96 and the temporal trend (NSE) was 0.96, demonstrating its accuracy. The census grazing data in 2015 was calculated according to the average sheep unit per pixel for each county, which poorly expressed the spatial heterogeneity within the counties (Fig. 4b, left figure). In contrast, the grazing spatialization dataset (Fig. 4b, right figure) considerably increased the within-county spatial heterogeneity while maintaining the original spatial distribution (R^2^ = 0.96, N = 242) (Fig. 4a). In addition, the grazing spatialization dataset exhibited consistent temporal trends with census grazing data at the regional level: the grazing intensity significantly increased from 1982 to 2015 (NSE = 0.96, N = 34) (Fig. 4c). Moreover, our dataset also can capture the spatial distribution of the temporal trends of census grazing data, that is, grazing intensity decreased and fluctuated in the central areas, but increased in others (Fig. 4d).

### Validation of the grazing spatialization dataset at the pixel level

**Fig. 5:**
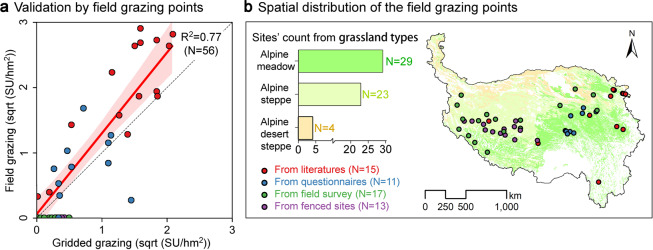
Validation of the grazing spatialization dataset using field grazing data: (**a**) validation by field grazing points; (**b**) Spatial distribution of the field grazing points.

To further validate the accuracy of our dataset, we collected field grazing data from 56 sites, and our dataset was highly consistent with the 56 field grazing data (R^2^ = 0.77). The data of the 56 sites were collected from the four aspects: data on 15 sites (red points) were obtained from 13 literatures on free grazing or conventional grazing (Table 4), data on 11 sites (blue points) and 17 sites (green points) were obtained from questionnaires and the field survey in August 2021 (no grazing), respectively, and data on other 13 sites (purple points) were obtained from fenced sites (no grazing)^50^.The unit of grazing intensity was transformed into standard sheep unit/ hm^2^ (SU/hm^2^) according to the Eq. (8). The grazing intensity of the 56 sites were ranged from 0 to 8.50 sheep unit per hectare (SU/hm^2^), and their distribution covered three grassland types: the alpine meadow (N = 29), alpine steppe (N = 23), and alpine desert steppe (N = 4). The spatial accuracy (R^2^) of the two datasets from different sources reached to 0.77 (N = 56, Fig. 5b), and it can make up for the uncertainty verified by census grazing data to a certain extent.

**Table 4:** Field grazing intensity (GI) data from literatures.

No.	Location (County)	Longitude (°E)	Latitude (°N)	Altitude (m)	GI (SU/hm^2^)
1	Menyuan	101.32	37.62	3200.00	3.75^56^
2	Haiyan	100.86	36.96	3130.00	5.00^57^
3	Menyuan	101.20–101.38	37.48–37.75	3250.00	3.51^58^
4	Tianzhu	102.12–103.77	36.52–37.92	2960.00	6.08^59^
5	Naqu	—	—	—	0.16^60^
6	Gaize	—	—	—	2.05^60^
7	Tianzhu	102.52	37.28	3600.00	1.64^61^
8	Maqu	101.85	33.67	3500.00	8.00^62^
9	Ruoergai	—	—	—	7.00^63^
10	Tianzhu	102.46	37.18	2960.00	7.00-8.00^64^
11	Shangri-La	99.60–99.98	27.33–27.72	3200.00–3400.00	3.50^65^
12	Menyuan	101.31	37.61	3212.00	7.50^66^
13	Maduo	—	—	—	0.12^67^
14	Menyuan	101.17	37.67	3241.00	7.25 ± 0.25^68^
15	Menyuan	101.18	37.67	3239.00	8.50 ± 0.35^68^

### Comparison with other grazing spatialization datasets

**Fig. 6:**
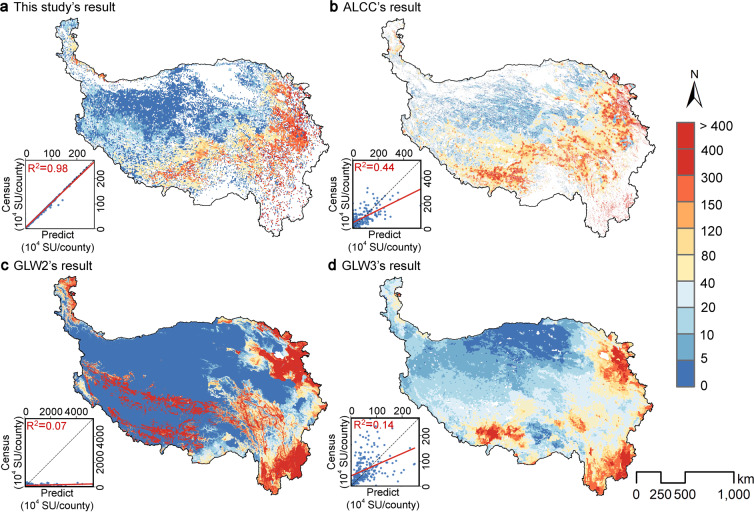
Comparisons of grazing spatial patterns for 2001: (**a**) this study result; (**b**) ALCC result; (**c**) GLW2 result; (**d**) GLW3 result. ALCC, GLW2, and GLW3 denote the actual livestock-carrying capacity, Gridded Livestock of the World 2.01, and Gridded Livestock of the World 3, respectively.

The temporal and spatial resolutions of our dataset are advantageous over the actual livestock carrying capacity (ALCC) dataset, Gridded Livestock of the World 2.01 (GLW2), and Gridded Livestock of the World 3 (GLW3) (Table 5) in the following aspects: The temporal resolution of our dataset (1982–2015) is higher than those of the three public datasets. Our dataset is more suitable for long-term scale research than ALCC (2000–2019), while the other two global livestock datasets (GLW2 and GLW3) have been proved unsuitable for long-term series studies^47^. In addition, our dataset can improve the accuracy of spatial resolution (Fig. 6). Considering that the census data of GLW2 and GLW3 are for 2001, the accuracies of the four datasets were compared for the corresponding year. The four datasets exhibited relatively consistent spatial patterns, showing high and low spatial patterns in the southeast and northwest, respectively. However, the datasets significantly differed in accuracy. Our dataset exhibited the highest accuracy (R^2^ = 0.98), which was at least twice those of the others, followed by ALCC (R^2^ = 0.44), and the global livestock datasets GLW2 and GLW3 exhibited poor accuracy, with R^2^ of 0.07 and 0.14, respectively.

**Table 5:** Summary of this study result and the results of other three public gridded grazing datasets on the Qinghai–Tibet Plateau.

Dataset		Spatial scale	Temporal resolution	Spatial resolution	Unit	Method	Livestock type	Data sources
This Study	Gridded Grazing Dataset	Regional	1982–2015	0.083°	SU/pixel	Improved RF model	Standard SU	10.6084/m9.figshare.21501390^49^
ALCC	Actual livestock-carrying capacity	Regional	2000–2019	250 m	SU/km^2^	Multiple linear regression	Standard SU	https://data.tpdc.ac.cn/zh-hans/^69^
GLW2	Gridded Livestock of the World 2.01	Global	2006	0.0083°	Heads/km^2^	Stratified linear multiple regressions	Cattle, ducks,	https://data.apps.fao.org/map/catalog/srv/eng/catalog.search#/home
							pigs, chickens,	
							sheep, goats	
GLW3	Gridded Livestock of the World 3	Global	2010	0.083°	Heads/pixel	RF model	Cattle, ducks,	http://www.fao.org/livestock-systems/
							pigs, chickens,	
							sheep, goats,	
							buffaloes, horses

### Uncertainties and limitations of grazing spatialization dataset

**Fig. 7:**
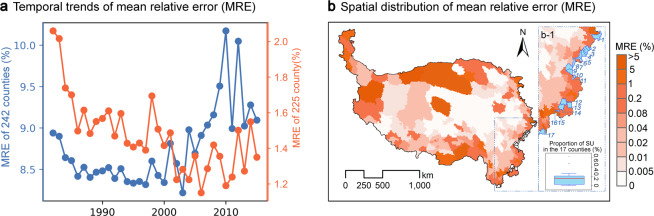
Uncertainties associated with grazing spatialization dataset: (**a**) Temporal trends of mean relative error (MRE); the orange and blue solid lines indicate the average errors for 252 and 242 counties in the corresponding year, respectively, and the difference between the data for the 252 and 242 counties was due to the lack of grassland boundary in the other 17 counties. (**b**) The spatial distribution of MRE for the 252 counties. The 17 counties are displayed in the light blue areas, and the proportion of SU in the 17 counties is summarized in b-1.

(i) Overall, the relative error associated with the dataset from 1982 to 2015 was 8.77%, calculated from the average errors of the 242 counties, which fluctuated with temporal trends (Fig. 7a, blue solid line). Among these counties, 225 counties exhibited a mean error of only 1.47% (Fig. 7a, orange solid line), attributable to the unreasonableness of our method, which is worth further improving in future studies. The error related to the other 17 counties originated from the incompleteness of auxiliary variables (Fig. 7b, bright blue areas); nonetheless, their influence on the Qinghai–Tibet Plateau is almost negligible because the 17 counties accounted for a mean SU of only 0.15% (Fig. 7b). (ii) In addition, our dataset might have some limitations. Estimating the dataset using the livestock stocking quantity at the end of year may result in the underestimation of grazing intensity, because the rates of livestock off-take were not considered owing to the data unavailability. Moreover, it’s should be clarified that proportion of forage-dependent livestock^51^, the distribution of nomadic herding^52^ and others, such as the regional differences in grazing time (equation S1), were not considered here.

## Supplementary information

Supplementary Table S1. The comparison of average theoretical livestock carrying capacity (LCCT) among our result and others on the Qinghai–Tibet Plateau

Supplementary information

Supplementary Figure S1. The theoretical livestock carrying capacity (LCCT)

## Data Availability

The code is fully operational under Python 3.6, and the Python scripts used to implement the gridded grazing dataset can be obtained from https://github.com/nanmeng123456/Grazing-spatilization.git. Further questions can be directed to Nan Meng (nanmeng_st@rcees.ac.cn).

## References

[1] Sun YX (2021). Grazing intensity and human activity intensity data sets on the Qinghai- Tibetan Plateau during 1990–2015. Geosci. Data J..

[2] Sun J (2022). Toward a sustainable grassland ecosystem worldwide. Innovation-Amsterdam..

[3] Bardgett RD (2021). Combatting global grassland degradation. Nat. Rev. Earth Environ..

[4] Dai LC (2021). Effect of grazing management strategies on alpine grassland on the northeastern Qinghai-Tibet Plateau. Ecol. Eng..

[5] O’Mara FP (2012). The role of grasslands in food security and climate change. Ann. Bot..

[6] Yu LF (2019). Effects of grazing exclusion on soil carbon dynamics in alpine grasslands of the Tibetan Plateau. Geoderma..

[7] Yang X (2020). Global negative effects of livestock grazing on arbuscular mycorrhizas: A meta-analysis. Sci. Total Environ..

[8] Odadi WO (2011). African wild ungulates compete with or facilitate cattle depending on season. Science..

[9] Li GY (2019). Grazing alters the phenology of alpine steppe by changing the surface physical environment on the northeast Qinghai-Tibet Plateau, China. J. Environ. Manage..

[10] Wei YQ (2022). Dual influence of climate change and anthropogenic activities on the spatiotemporal vegetation dynamics over the Qinghai-Tibetan Plateau from 1981 to 2015. Earth’s Future..

[11] Pozo RA (2021). Reconciling livestock production and wild herbivore conservation: challenges and opportunities. Trends Ecol. Evol..

[12] Robinson TP (2014). Mapping the global distribution of livestock. Plos One..

[13] Nicolas G (2016). Using random forest to improve the downscaling of global livestock census data. Plos One..

[14] Ren JZ (2012). Grazing, the basic form of grassland ecosystem and its transformation. Journal of Natural Resources..

[15] Homburger H (2015). Patterns of livestock activity on heterogeneous subalpine pastures reveal distinct responses to spatial autocorrelation, environment and management. Mov. Ecol..

[16] Rivero MJ (2021). Factors affecting site use preference of grazing cattle studied from 2000 to 2020 through GPS tracking: a review. Sensors..

[17] Halasz A (2016). Weather regulated cattle behaviour on rangeland. Appl. Ecol. Environ. Res..

[18] Tomkins N, O’Reagain P (2007). Global positioning systems indicate landscape preferences of cattle in the subtropical savannas. Rangeland J..

[19] Ganskopp D (2001). Manipulating cattle distribution with salt and water in large arid-land pastures: a GPS/GIS assessment. Appl. Anim. Behav. Sci..

[20] Hu XY (2022). Spatialization method of grazing intensity and its application in Tibetan Plateau. Acta Geographica Sinica..

[21] Wu RD (2014). Optimized spatial priorities for biodiversity conservation in China: a systematic conservation planning perspective. Plos One..

[22] Ma CH (2022). Spatial quantification method of grassland utilization intensity on the Qinghai-Tibetan Plateau: A case study on the Selinco basin. J. Environ. Manage..

[23] Kastner T (2021). Land use intensification increasingly drives the spatiotemporal patterns of the global human appropriation of net primary production in the last century. Glob. Change Biol..

[24] Ren YH (2021). Optimizing livestock carrying capacity for wild ungulate-livestock coexistence in a Qinghai-Tibet Plateau grassland. Sci Rep..

[25] Gilbert M (2018). Global distribution data for cattle, buffaloes, horses, sheep, goats, pigs, chickens and ducks in 2010. Sci. Data..

[26] Mei Y (2022). Population spatialization with pixel-level attribute grading by considering scale mismatch issue in regression modeling. Geo-Spat. Inf. Sci..

[27] Gaughan AE (2016). Spatiotemporal patterns of population in mainland China,1990 to 2010. Sci. Data..

[28] Liang HD (2020). GDP spatialization in Ningbo city based on NPP/VIIRS night-time light and auxiliary data using random forest regression. Advance in Space Research..

[29] Fararoda R (2021). Improving forest above ground biomass estimates over Indian forests using multi source data sets with machine learning algorithm. Ecol. Inform..

[30] Breiman L (2001). Random forests. Mach. Learn..

[31] Dong SK (2020). Enhancing sustainability of grassland ecosystems through ecological restoration and grazing management in an era of climate change on Qinghai-Tibetan Plateau. Agriculture, Ecosystems and Environment..

[32] Li T (2022). Characteristics and trends of grassland degradation research. J. Soils Sediments..

[33] Gang CC (2014). Quantitative assessment of the contributions of climate change and human activities on global grassland degradation. Environ. Earth Sci..

[34] Meng N (2021). Climate change indirectly enhances sandstorm prevention services by altering ecosystem patterns on the Qinghai-Tibet Plateau. Journal of Monuntain Science..

[35] Fassnacht FE (2019). A Landsat-based vegetation trend product of the tibetan Plateau for the time-period 1990–2018. Sci. Data..

[36] Yang MY (2021). Trade-offs in ecological, productivity and livelihood dimensions inform sustainable grassland management: Case study from the Qinghai-Tibetan Plateau. Agriculture, Ecosystems and Environment..

[37] Huang W, Bruemmer B, Huntsinger L (2016). Incorporating measures of grassland productivity into efficiency estimates for livestock grazing on the Qinghai-Tibetan Plateau in China. Ecol. Econ..

[38] Jung M (2020). A global map of terrestrial habitat types. Sci. Data..

[39] Wang CH (2018). The construction and management of China’s nature reserves in the past forty years of reformand opening-up: achievements, challenges and prospects. Chinese Rural Economy..

[40] Sinha P (2019). Assessing the spatial sensitivity of a random forest model: application in gridded population modeling. Computers, Environment and Urban Systems..

[41] Wu, J. H. *et al*. Population Spatialization by Considering Pixel-level Attribute Grading and Spatial Association. *Geomatics and Information Science of Wuhan University*. 1–14, 10.13203/j.whugis20200379 (2021).

[42] Briggs DJ (2007). Dasymetric modelling of small-area population distribution using land cover and light emissions data. Remote Sens. Environ..

[43] Su MD (2010). Multi-layer multi-class dasymetric mapping to estimate population distribution. Sci. Total Environ..

[44] Stevens FR (2015). Disaggregating census data for population mapping using random forests with remotely-sensed and ancillary data. Plos One..

[45] Chen YY (2018). A new downscaling-integration framework for high-resolution monthly precipitation estimates: Combining rain gauge observations, satellite-derived precipitation data and geographical ancillary data. Remote Sens. Environ..

[46] Chen SD (2019). Spatial downscaling methods of soil moisture based on multisource remote sensing data and its application. Water..

[47] Li XH, Hou JL, Huang CL (2022). High-resolution gridded livestock projection for western China based on machine learning. Remote Sens..

[48] Wang, L. J. Ecological carrying capacity and changes in the Qinghai-Tibet Plateau. *Beijing: University of Chinese Academy of Sciences*. (In Chinese) (2022).

[49] Meng N (2022). figshare.

[50] Zhang X, Niu B (2019). National Tibetan Plateau Data Center.

[51] Li G (2014). Balance between actual number of livestock and livestock carrying capacity of grassland after added forage of straw based on remote sensing in Tibetan Plateau. Transactions of the Chinese Society of Agricultural Engineering..

[52] Zhuang MH (2019). Community-based seasonal movement grazing maintains lower greenhouse gas emission intensity on Qinghai-Tibet Plateau of China. Land Use Pol..

[53] He KD, Sun J, Chen QJ (2019). Response of climate and soil texture to net primary productivity and precipitation-use efficiency in the Tibetan Plateau. Pratacultural Science..

[54] Dai YJ (2013). Development of a China Dataset of Soil Hydraulic Parameters Using Pedotransfer Functions for Land Surface Modeling. J. Hydrometeorol..

[55] Shangguan. W., Dai. YJ. A China Dataset of soil hydraulic parameters pedotransfer functions for land surface modeling (1980). *National Tibetan Plateau/Third Pole Environment Data Center*. 10.11888/Soil.tpdc.270606 (2013).

[56] Dai LC (2021). Long-term grazing exclusion greatly improve carbon and nitrogen store in an alpine meadow on the northern Qinghai-Tibet Plateau. Catena..

[57] Chen J (2018). Divergent responses of ecosystem respiration components to livestock exclusion on the Qinghai Tibetan Plateau. Land Degrad. Dev..

[58] Zou JR (2016). Relationship of plant diversity with litter and soil available nitrogen in an alpine meadow under a 9-year grazing exclusion. Ecol. Res..

[59] Li W (2015). Analysis of soil respiration under different grazing management patterns in the alpine meadow-steppe of the Qinghai - Tibet Plateau. Acta Prataculturae Sinica..

[60] Lu X (2015). Short-term grazing exclusion has no impact on soil properties and nutrients of degraded alpine grassland in Tibet, China. Solid Earth..

[61] Shi XM (2013). Grazing exclusion decreases soil organic C storage at an alpine grassland of the Qinghai–Tibetan Plateau. Ecol. Eng..

[62] Wang X (2018). Grazing induces direct and indirect shrub effects on soil nematode communities. Soil Biology and Biochemistry..

[63] Zhang MH (2019). Community-based seasonal movement grazing maintains lower greenhouse gas emission intensity on Qinghai-Tibet Plateau of China. Land Use Pol..

[64] Wang JL (2020). Effects of grazing exclusion on soil respiration components in an alpine meadow on the north-eastern Qinghai-Tibet Plateau. Catena..

[65] Ma L (2021). Grazing rest versus no grazing stimulates soil inorganic N turnover in the alpine grasslands of the Qinghai-Tibet plateau. Catena..

[66] Guo XW (2021). Restoration of Degraded Grassland Significantly Improves Water Storage in Alpine Grasslands in the Qinghai-Tibet Plateau. Front. Plant Sci..

[67] Shao QQ (2018). Using UAV remote sensing to analyze the population and distribution of large wild herbivores. Journal of Remote Sensing..

[68] Lin L (2022). Response and adaptation of plant community in alpine kobresia meadow to different grazing intensities. Chinese Journal of Grassland..

[69] Liu BT (2021). Actual livestock carrying capacity estimation product in Qinghai-Tibet Plateau (2000-2019). National Tibetan Plateau Data Center..

