# Supplementary material for: A high-resolution gridded grazing dataset of grassland ecosystem on the Qinghai–Tibet Plateau in 1982–2015
Source: Sci Data. 2023 Feb 2;10:68. doi: 10.1038/s41597-023-01970-1 (PMC9895079; doi:10.1038/s41597-023-01970-1)
Supplement: Supplementary file 2 — Supplementary information [file 41597_2023_1970_MOESM2_ESM.docx]

Supporting Information for

**A high-resolution gridded grazing dataset of grassland ecosystem on the Qinghai–Tibet Plateau in 1982–2015**

Nan Meng^1,2^，Lijing Wang^1^, Wenchao Qi^3^, Xuhuan Dai^1^, Zuzheng Li^1^, Yanzheng Yang^1,2✉^ , Ruonan Li^1,2^, Jinfeng Ma^1,2^, Hua Zheng^1,2^

^1^ State Key Laboratory of Urban and Regional Ecology, Research Center for Eco-Environmental Sciences, Chinese Academy of Sciences, Beijing, 100085, China

^2^ University of Chinese Academy of Sciences, Beijing, 100049, China

^3^ Aerospace Information Research Institute, Chinese Academy of Sciences, Beijing, 100101, China.

**Methods for calculating theoretical livestock carrying capacity:**

The theoretical livestock carrying capacity is the suitable sheep unit for grazing per unit area of grassland. It is expressed in equation (S1) ^1^:

| ${LCC}_{T}=\frac{\frac{{NPP}_{P}}{C*\left( 1+{RS}_{r} \right)*\left( 1-{WC}_{r} \right)}*{PU}_{r}*E_{r}}{S*T}$ | (S1) |
| --- | --- |

where ${LCC}_{T}$ is the theoretical livestock carrying capacity (SU/hm^2^); ${NPP}_{P}$ is the potential net primary production (gC / m^2^ / year);$C$ is the conversion coefficient between biomass and carbon (0.45 gC/g); ${RS}_{r}$ is the root shoot ratio, with values for alpine meadow and alpine steppe as 6.80 and 5.20, respectively; ${WC}_{r}$ is the water content ratio (15%). ${PU}_{r}$ is the proper utilization rate (0.85); $E_{r}$ is edible ratio of the hay, with values for alpine meadow and alpine steppe as 0.76 and 0.69, respectively; S is the daily feed hay for the standardized sheep unit (1.33 kg/SU/days); and $T$ is the grazing time in each year (365 days).

To estimate the potential net primary productivity, the simulation process based on the RF model was as follows: (a) Obtaining the dependent variable and independent variables: The dependent variable was the net primary production^2^, while the independent variables included three climate variables (precipitation, temperature, and radiation) in growing season (May to September in each year), five soil variables (soil moisture, pH, available N, available P, available K), and three topographical factors (DEM, slope, aspect); the information of the variables is presented in Table 3. (b) Training the RF model: The training samples were extracted from the samples of the top 90% NPP of each ecoregion and grassland type combination, and the RF model was trained and tested according to these sample. The training and testing samples were divided in the proportion of 3:1 (c) Estimating the potential NPP: The trained RF model was applied to all grassland pixels to obtain the spatial distribution of the potential NPP driven by independent variables. (d) Resampling the potential NPP: The spatial resolution of potential NPP was resampled to 0.083°, as the with NDVI dataset.

**Results of theoretical livestock carrying capacity:**

The average theoretical livestock carrying capacity on the Qinghai–Tibet Plateau was 0.56 SU/hm^2^ for 2001-2019 in our study, and the results were reliable compared with others in the literatures (Table S1). The spatial patterns of the theoretical livestock carrying capacity on the Qinghai–Tibet Plateau showed that it gradually decreased from southeast to northwest (Figure S1a). The alpine meadow, alpine steppe, and alpine desert steppe are the main vegetation type groups on the Qinghai Tibet Plateau according to the vegetation map of the People's Republic of China (1:1000000)^3^, and other vegetation type groups within grassland with a small proportion were merged into the three grassland types (Figure 1a). The theoretical livestock carrying capacity for the three grassland types were calculated by their average value, namely 0.43 SU/hm^2^ for alpine meadow, 0.35 SU/hm^2^ for alpine steppe, and 0.21 SU/hm^2^ for alpine desert steppe (Figure S1b). Therefore, 0.43 SU/hm^2^, 0.35 SU/hm^2^, and 0.21 SU/hm^2^ were considered as the thresholds for the three grassland types in the step 3 of our methodological framework, respectively.

Table S1. The comparison of average theoretical livestock carrying capacity (LCC_T_) among our result and others on the Qinghai–Tibet Plateau

| **No.** | **Average LCC_T_** | **Period** |
| --- | --- | --- |
| 1 | **0.56 SU/hm^2^** (our result) | 2001-2019 |
| 2 | 0.49 SU/hm^2 4^ | 2003–2004 |
| 3 | 0.94 SU/hm^2 5^ | 2009–2017 |
| 4 | 0.40-0.50 SU/hm^2 6^ | 2000-2018 |
| 5 | 0.60 SU/hm^2 7^ | 2015 |


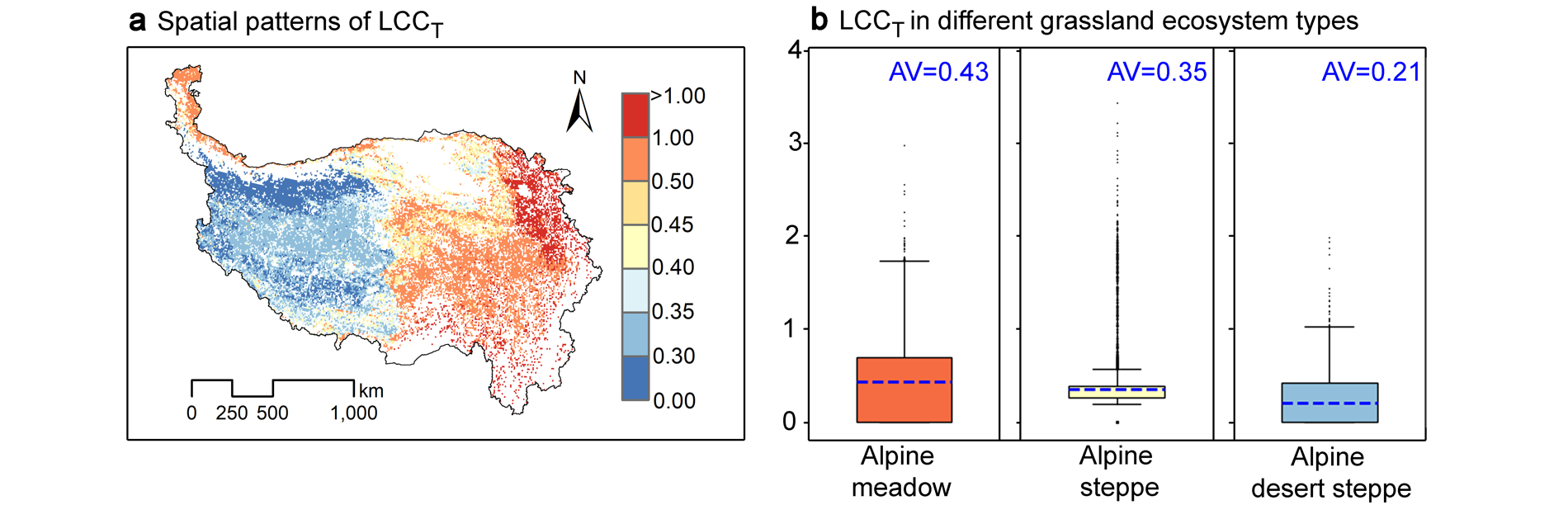


Figure S1. The theoretical livestock carrying capacity (LCC_T_): (a) Spatial patterns of LCC_T_, and (b) LCC_T_ in different grassland ecosystem types. AV is average value of the LCC_T_, which is represented by the blue dashed line in the boxplots.

**Other information**

The main datasets used to construct Figure 1 were as follows: Grassland types were displayed according to the vegetation map of the People's Republic of China (1:1000000)^3^, provided by the National Cryosphere Desert Data Center (<http://www.ncdc.ac.cn>). Grassland production was represented by the net primary production, obtained from the National Earth System Science Data Center of China (<http://www.geodata.cn/>)^2^. Grazing density and economic development were in terms of the actual livestock-carrying capacity^8^ and human footprint^9^, obtained from the National Tibetan Plateau Data Center (<https://data.tpdc.ac.cn/zh-hans/>).

**References**

1. Mo. XG., Liu. W., Meng. CC., et.al. Variations of forage yield and forage-livestock balance in grasslands over the Tibetan Plateau, China. *Chinese Journal of Applied Ecology*. **32**, 2415-2425, https://doi.org/10.13287/j.1001-9332.202107.002 (2021). (In Chinese).

2. Wang. J., Sun. R., Zhang. H., et.al. New Global MuSyQ GPP/NPP Remote Sensing Products From 1981 to 2018. *Ieee J. Sel. Top. Appl. Earth Observ. Remote Sens.* **14**, 5596-5612, https://doi/https://doi/10.1109/JSTARS.2021.3076075 (2021).

3. Zhang. XS. Vegetation Map of the People's Republic of China (1:1000000). *Beijing:GeologicalPublishingHouse*. (2007). (In Chinese).

4. Qian. S., Mao. LX., Hou. YY., et.al. Livestock carrying capacity and balance between carrying capacity of grassland with added forage and actual livestock in the Qinghai-Tibet Plateau. *Journal of Natural Resources*. **22**, 389-397+498, https://doi.org/10.3321/j.issn:1000-3037.2007.03.008 (2007). (In Chinese).

5. Zhang. XZ., Li. M., Wu. JS., et.al. Alpine grassland aboveground biomass and theoretical livestock carrying capacity on the Tibetan Plateau. *Journal of Resources and Ecology*. **13**, 129-141, https://doi.org/10.5814/j.issn.1674-764x.2022.01.015 (2022).(In Chinese).

6. Wang. LJ, Xiao. Y, Kong. LQ., et.al. Spatiotemporal patterns and early-warning of grassland carrying capacity in the Qinghai-Tibet Plateau. *Acta Ecologica Sinica*. **42**, 6684-6694, (2022). (In Chinese).

7. Wang. XQ. Analysis on the temporal and spatial changes of grazing activities and the balance between forage and livestock in the Qinghai-Tibet Plateau from 2000 to 2018. *Xi'an: Chang'an University*. (2021). (In Chinese).

8. Liu. BT. Actual livestock carrying capacity estimation product in Qinghai-Tibet Plateau (2000-2019). *National Tibetan Plateau Data Center*. https://doi.org/10.11888/Ecolo.tpdc.271513 (2021).

9. Duan. Q., Luo. L. A dataset of human footprint over the Qinghai-Tibet Plateau during 1990–2017. *National Tibetan Plateau Data Center*. https://doi.org/10.11922/sciencedb.933 (2021).
